# Alopecia areata patients are associated with increased risk of glaucoma: a multicenter, retrospective cohort study

**DOI:** 10.3389/fimmu.2026.1870857

**Published:** 2026-08-12

**Authors:** Hui-Chin Chang, Vincent Ping-Sheng Lai, Yu-Jung Su, Shiu-Jau Chen, Meng-Che Wu, Shuo-Yan Gau

**Affiliations:** 1 Evidence-based Medicine Center, Chung Shan Medical University Hospital, Taichung, Taiwan; 2 Library, Chung Shan Medical University Hospital, Taichung, Taiwan; 3 School of Medicine, Chung Shan Medical University, Taichung, Taiwan; 4 Orthopedics Department, Chi-Mei Medical Center, Tainan, Taiwan; 5 Department of Neurosurgery, MacKay Memorial Hospital, Taipei, Taiwan; 6 Department of Medicine, MacKay Medical University, New Taipei, Taiwan; 7 Division of Pediatric Gastroenterology, Children’s Medical Center, Taichung Veterans General Hospital, Taichung, Taiwan; 8 Department of Post-Baccalaureate Medicine, College of Medicine, National Chung Hsing University, Taichung, Taiwan; 9 Department and Graduate Institute of Business Administration, National Taiwan University, Taipei, Taiwan; 10 Institute of Allergology, Charité-Universitätsmedizin Berlin, Corporate Member of Freie Universität Berlin and Humboldt-Universität zu Berlin, Berlin, Germany; 11 Department of Medical Education, Ditmanson Medical Foundation Chia-Yi Christian Hospital, Chiayi, Taiwan

**Keywords:** alopecia areata, cohort, glaucoma, real world evidence, TriNetX

## Abstract

**Background:**

Alopecia areata (AA) is a T cell–mediated autoimmune disease characterized by systemic immune activation that may extend to ocular tissues. However, whether AA increases the long-term risk of glaucoma remains unclear.

**Methods:**

We conducted a multicenter retrospective cohort study using the TriNetX Global Collaborative Network, comprising over 144 million patients across 147 healthcare organizations. For cross-database validation, we additionally performed a parallel analysis in another dataset, the US collaborative network, restricting the population to patients from healthcare organizations in the United States. Adults (≥18 years) with at least two visit records and a diagnosis of AA between 2005 and 2023 were included and matched 1:1 with individuals undergoing general health examinations without an AA diagnosis. Exclusion criteria included prior glaucoma, malignancy, or death before the index date. Propensity score matching incorporated demographic, socioeconomic, and clinical factors, including hypertension, diabetes, conjunctivitis, and Sjögren syndrome. The primary endpoint was new-onset glaucoma, with additional assessment of subtypes. Stratified analyses were performed by age and sex, and sensitivity analyses applied alternative definitions, matching models, and washout periods. Hazard ratios (HRs) and 95% confidence intervals (CIs) were estimated.

**Results:**

The final matched cohort included 28,966 patients with AA and 28,966 controls. During a 15-year follow-up, AA was associated with a higher risk of glaucoma (HR = 1.89; 95% CI, 1.51–2.38), consistent across models and sensitivity analyses. The risk was elevated for primary glaucoma (HR = 1.99; 95% CI, 1.10–3.62) and glaucoma-suspect states. Stratified analyses revealed stronger associations in females (HR = 1.78; 95% CI, 1.36–2.33) and older adults (≥65 years; HR = 2.76; 95% CI, 1.95–3.89). For the cross-database validation, the association remained significant in the US collaborative network, with a hazard ratio of 2.11 (95% CI, 1.72–2.59).

**Conclusions:**

AA was associated with a higher long-term risk of glaucoma across the primary, sensitivity, and cross-database analyses. However, this observational study establishes an epidemiologic association only and does not demonstrate a direct biological or immune-mediated link. Further prospective studies incorporating detailed ophthalmic examinations, longitudinal intraocular pressure measurements, retinal imaging, and systemic or ocular immune biomarkers are needed to determine whether shared inflammatory pathways contribute to this association.

## Introduction

Alopecia areata (AA) is a prototypical T cell–mediated autoimmune disorder characterized by nonscarring hair loss resulting from the collapse of hair follicle immune privilege (1, 2). Real-world evidence indicates that AA is associated with systemic inflammatory diseases in different organ systems (3–5), and emerging data further suggest systemic immune involvement, characterized by heightened Th1/Th17 activity and expansion of cytotoxic CD8^+^ T cells, which may extend beyond cutaneous manifestations to other immune-privileged sites, particularly the ocular compartment (6–8).

Glaucoma has been attributed to progressive optic nerve degeneration secondary to elevated intraocular pressure (IOP). However, population-based analyses have reported a significantly increased systemic inflammatory response index (SIRI) in patients with glaucoma (9). Chronic inflammation and infection have also been proposed as potential risk factors for glaucoma in previous real-world studies (10, 11). Mechanistic studies also show that these patients exhibit robust autoreactive T-cell responses, including increased heat shock protein (HSP)–reactive T cells, supporting an autoimmune contribution to glaucomatous optic neuropathy (12).

Previous studies have indicated a greater prevalence of autoimmune diseases among patients with primary open-angle glaucoma (POAG) (13). In parallel, several studies have shown that patients with AA often present with ophthalmic abnormalities. A systematic review including 87 studies reported higher rates of ocular abnormalities in AA than in the general population, supporting broader ocular vulnerability in this group (14–16).

Although associations between AA and nonspecific ocular inflammation, such as uveitis, conjunctivitis, and lacrimal dysfunction, have been documented in retrospective cohorts and meta-analyses (7, 15), the long-term risk of glaucomatous optic neuropathy in patients with AA remains uncharacterized. Existing studies are limited by single-center or cross-sectional designs and insufficient follow-up duration, precluding definitive inference regarding AA’s role in glaucoma pathogenesis. To address these gaps, we conducted a multicenter retrospective cohort study utilizing the TriNetX global electronic health record network to evaluate the association between AA and the subsequent risk of new-onset glaucoma.

## Materials and methods

Clinical information for this retrospective cohort study was obtained from the TriNetX global federated research platform, an international repository of de-identified electronic health records contributed by multiple healthcare systems and widely used across diverse fields of health outcomes research (17–19). For the main analysis, we applied the Global Collaborative Network, which integrates data from 147 participating institutions across North and South America, Europe, and Asia and includes records from more than 144 million unique patients. For cross-database validation, we additionally performed a parallel analysis in another subset, the US collaborative network, restricting the population to patients from healthcare organizations in the United States. The study protocol was reviewed and approved by the Institutional Review Board of Chung Shan Medical University Hospital (IRB No. CS1-25002) and Chi Mei Medical Center (IRB No. 11502-E02) and was conducted in accordance with the Declaration of Helsinki.

The AA cohort included adults with at least two separate healthcare encounters and a recorded diagnosis of AA between January 1, 2005, and December 31, 2023. The index date was defined as the date of the qualifying AA diagnosis. The non-AA control cohort was drawn from individuals who underwent a routine general health examination during the same study period, identified using ICD-10-CM code Z00. For controls, the index date was defined as the date of the qualifying Z00-coded encounter. Individuals in the control cohort were required to have no recorded diagnosis of AA before or on the index date. The same eligibility criteria were applied to both cohorts. Individuals younger than 18 years, those with any history of malignancy, those documented as deceased, and those with glaucoma diagnosed before or on the index date were excluded. Also, both cohorts were restricted to individuals with at least two recorded visits to reduce inclusion of patients with insufficient longitudinal information. Eligible individuals were subsequently matched 1:1 using the prespecified propensity score model. Propensity scores incorporated sociodemographic and clinical determinants, including age, sex, race, body mass index, mental and behavioral disorders related to psychoactive substance use, markers of adverse socioeconomic or psychosocial conditions, and relevant comorbidities (hypertension, hyperlipidemia, diabetes mellitus, conjunctivitis, iridocyclitis, and Sjögren syndrome). To partially account for differences in healthcare utilization, available proxies, including prior inpatient encounters and emergency department visits, were incorporated into the propensity score matching model. Covariates were selected *a priori* based on their plausible relationships with the outcome event. Demographic factors were included because the epidemiology of both diseases varies by age, sex, and race, whereas body mass index and cardiometabolic comorbidities were included to account for metabolic and vascular glaucoma risk. Conjunctivitis, iridocyclitis, and Sjögren syndrome were incorporated because ocular inflammation and autoimmune disease may influence both ophthalmic surveillance and glaucoma development (20). Mental and behavioral disorders related to psychoactive substance use and socioeconomic or psychosocial circumstances indicators were included due to their potential influence on detection of glaucoma risk (21–23). Detailed information on visit frequency or ophthalmic examination intensity was not available within the TriNetX platform.

The primary endpoint was new-onset glaucoma-related diagnostic coding, defined using standardized International Classification of Diseases, Tenth Revision, Clinical Modification (ICD-10-CM) codes (H40–H42). Subtype analyses were conducted based on predefined diagnostic categories, including primary glaucoma (H40.11, H40.2, Q15.0), secondary glaucoma (H40.3–H40.6), open-angle glaucoma (H40.1), angle-closure glaucoma (H40.2), and glaucoma-suspect conditions (H40.0). Additional subclassifications included preglaucoma (H40.00), open-angle glaucoma with borderline findings (H40.01–H40.02), anatomical narrow angle (H40.03), and ocular hypertension (H40.05). Because intraocular pressure measurements, optical coherence tomography findings, visual-field parameters, optic-disc assessments, and specialist adjudication were not available, these outcomes represent administrative diagnostic coding rather than glaucoma confirmed according to standardized ophthalmologic criteria. Follow-up commenced at the index date and continued until the diagnosis of glaucoma or the last available record, whichever occurred first.

To evaluate the stability of the findings and minimize residual confounding, we performed several sensitivity analyses, including alternative matching specifications, revised operational definitions of exposures and outcomes, and washout periods designed to reduce reverse causality. Subgroup analyses stratified by age and sex were conducted to examine potential effect modification. To further address potential confounding by corticosteroid exposure, we conducted additional analyses, including (1) a sensitivity analysis excluding individuals with systemic corticosteroid use and (2) an alternative propensity score–matching model incorporating systemic corticosteroid use as a covariate.

To further address potential surveillance bias, we conducted an active-comparator analysis using patients with psoriasis. The AA cohort was defined according to the same criteria as in the primary analysis. The reference cohort consisted of individuals with psoriasis, identified using ICD-10-CM code L40, without any diagnostic coding indicative of AA. The same exclusion criteria were applied to both cohorts: age younger than 18 years, any history of malignancy, a recorded death status, and glaucoma diagnosed before or on the index date. The index date was defined as the date of the qualifying AA or psoriasis diagnosis. The AA and psoriasis cohorts were matched 1:1 using propensity scores based on the same sociodemographic and clinical covariates applied in the primary analysis, including age, sex, race, body mass index, mental and behavioral disorders related to psychoactive substance use, markers of adverse socioeconomic or psychosocial circumstances, hypertension, hyperlipidemia, diabetes mellitus, conjunctivitis, iridocyclitis, and Sjögren syndrome. Prior inpatient encounters and emergency department visits were additionally included as available proxies for healthcare utilization. The primary outcome in this active-comparator analysis was new-onset glaucoma defined by ICD-10-CM codes H40–H42.

All statistical analyses were conducted within the TriNetX analytics interface. Hazard ratios (HRs) and 95% confidence intervals (CIs) were calculated to compare the risk of glaucoma between the AA and control cohorts. Post-matching covariate balance was assessed using standardized mean differences, with values below 0.1 considered indicative of adequate balance in baseline characteristics.

## Results

### Baseline characteristics

**Figure 1:**
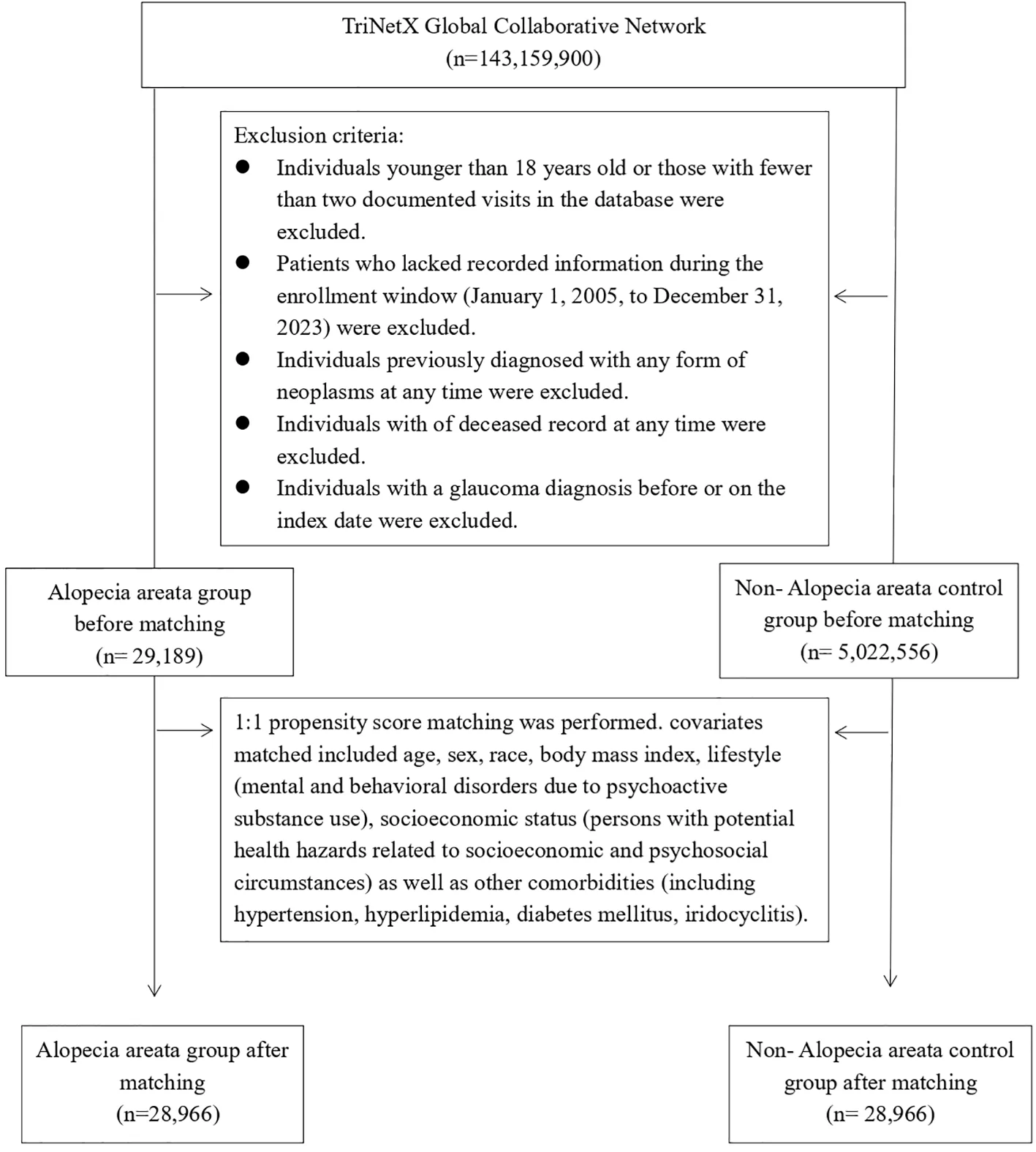
Patient selection process.

**Figure 1** shows the baseline characteristics of the study population. A total of 28,966 patients with alopecia areata (AA) were matched 1:1 with controls through propensity score methods. The mean age in the AA group was 33.0 years, and 57.8% of patients were female (**Table 1**). Prior to matching, notable differences were observed in age, as well as in the prevalence of hypertension and conjunctivitis, between the AA and control groups. After matching, these imbalances were reduced, and baseline covariates became broadly comparable across demographic and clinical variables.

**Table 1:** Baseline characteristics .

	Before matching			After matching^a^		
	AA cohort (n= 29,189)	Control cohort (n=5,022,556)	SMD	AA cohort (n=28,966)	Control cohort (n=28,966)	SMD
Age at index						
Mean ± SD	33.0 ± 16.7	39.2 ± 20.2	0.34	33.0 ± 16.7	33.0 ± 16.7	0.00
Sex						
Female	16733 (57.8)	2782968 (55.6)	0.04	16733 (57.8)	16722 (57.7)	0.00
Male	12207 (42.1)	2195448 (43.9)	0.04	12207 (42.1)	12105 (41.8)	0.01
Unknown Gender	26 (<0.1)	25085 (0.5)	0.08	26 (<0.1)	139 (0.5)	0.07
Race, n (%)						
White	11406 (39.4)	2441355 (48.8)	0.19	11406 (39.4)	11399 (39.4)	0.00
Black or African American	4577 (15.8)	697954 (13.9)	0.05	4577 (15.8)	5146 (17.8)	0.05
Asian	3070 (10.6)	250510 (5.0)	0.21	3070 (10.6)	1777 (6.1)	0.16
Native Hawaiian or Other Pacific Islander	247 (0.9)	29288 (0.6)	0.03	247 (0.9)	253 (0.9)	0.00
American Indian or Alaska Native	160 (0.6)	16072 (0.3)	0.04	160 (0.6)	116 (0.4)	0.02
Other Race	1784 (6.2)	242131 (4.8)	0.06	1784 (6.2)	1670 (5.8)	0.02
Unknown Race	7722 (26.7)	1326191 (26.5)	0.00	7722 (26.7)	8605 (29.7)	0.07
Body mass index (BMI)						
Greater than 25 kg/m^2^, n (%)	5530 (19.1)	1023327 (20.5)	0.03	5530 (19.1)	5525 (19.1)	0.00
Socioeconomic status						
Persons with potential health hazards related to socioeconomic and psychosocial circumstances	431 (1.5)	62419 (1.2)	0.02	431 (1.5)	425 (1.5)	0.00
Lifestyle						
Mental and behavioral disorders due to psychoactive substance use	1628 (5.6)	229334 (4.6)	0.05	1628 (5.6)	1626 (5.6)	0.00
Comorbidities						
Essential hypertension	2139 (7.4)	547940 (11.0)	0.12	2139 (7.4)	2124 (7.3)	0.00
Hyperlipidemia, unspecified	1437 (5.0)	306227 (6.1)	0.05	1437 (5.0)	1423 (4.9)	0.00
Conjunctivitis	1193 (4.1)	93085 (1.9)	0.13	1193 (4.1)	1194 (4.1)	0.00
Diabetes mellitus	782 (2.7)	198760 (4.0)	0.07	782 (2.7)	787 (2.7)	0.00
Systemic lupus erythematosus	268 (0.9)	8491 (0.2)	0.10	268 (0.9)	77 (0.3)	0.09
Chronic ischemic heart disease	253 (0.9)	95405 (1.9)	0.09	253 (0.9)	250 (0.9)	0.00
Sjögren syndrome	124 (0.4)	6599 (0.1)	0.06	124 (0.4)	129 (0.4)	0.00
Rheumatoid arthritis	54 (0.2)	5806 (0.1)	0.02	54 (0.2)	28 (0.1)	0.02
Iridocyclitis	45 (0.2)	3907 (0.1)	0.02	45 (0.2)	33 (0.1)	0.01
Ankylosing spondylitis	36 (0.1)	2798 (0.1)	0.02	36 (0.1)	19 (0.1)	0.02
Comedications						
Corticosteroids for systemic use	6994 (24.1)	802597 (16.0)	0.20	6994 (24.1)	4705 (16.2)	0.20
Epinephrine	1298 (4.5)	157640 (3.2)	0.07	1298 (4.5)	895 (3.1)	0.07
Anticholinergics	616 (2.1)	115118 (2.3)	0.01	616 (2.1)	566 (2.0)	0.01
Tricyclic antidepressants	540 (1.9)	56576 (1.1)	0.06	540 (1.9)	304 (1.1)	0.07

AA, alopecia areata; SMD, standardized mean difference; SD, Standardized difference.

^a^ 1:1 propensity score matching was performed. covariates matched included age, sex, race, BMI, lifestyle (mental and behavioral disorders due to psychoactive substance use), socioeconomic status (persons with potential health hazards related to socioeconomic and psychosocial circumstances) as well as other comorbidities (including hypertension, hyperlipidemia, diabetes mellitus, iridocyclitis).

### Risk of glaucoma in patients with alopecia areata

**Figure 2:**
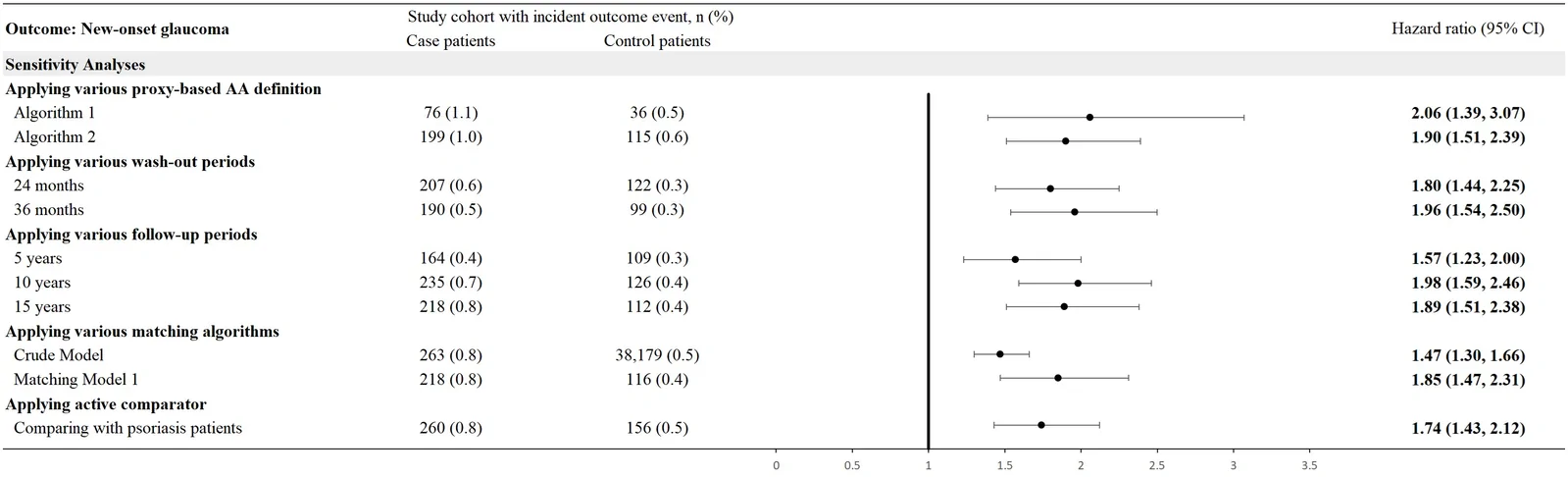
Risk of glaucoma in AA patients in different sensitivity models.

**Figure 2** summarizes the long-term risk of new-onset glaucoma in patients with AA. At 5 years of follow-up, patients with AA presented a 1.57-fold higher incidence of new-onset glaucoma (95% CI: 1.23–2.00). This risk remained elevated over longer follow-up durations, including 10 years (HR: 1.98; 95% CI: 1.59–2.46) and 15 years (HR: 1.89; 95% CI: 1.51–2.38). Given the potential confounding effect of corticosteroid exposure, additional analyses were performed. After excluding individuals with systemic corticosteroid use, the association between AA and glaucoma remained statistically significant. Similarly, an alternative propensity score matching model incorporating systemic corticosteroid use as a covariate yielded consistent results (**Supplementary Table 3**). In the active-comparator analysis using patients with psoriasis, AA remained associated with a higher risk of glaucoma than psoriasis (HR, 1.74; 95% CI, 1.43–2.12). Although the magnitude of association was modestly attenuated compared with the primary analysis using the general examination cohort, the direction and statistical significance of the association remained (**Figure 2**). The Kaplan–Meier curves in **Supplementary Figure 1** further illustrated an early and sustained divergence in cumulative glaucoma incidence between cohorts, with separation evident within the first 1–2 years of follow-up and persisting throughout the 15-year observation period. To further validate the association between AA and glaucoma, **Supplementary Figure 2** presents the results of applying outcome of glaucoma excluding angle closure and secondary glaucoma, with the significance remained. In an additional sensitivity analysis excluding glaucoma-suspect diagnoses (H40.0) from the composite outcome, AA remained associated with a higher risk of subsequently recorded glaucoma diagnoses (HR, 1.68; 95% CI, 1.01–2.78; **Supplementary Figure 3**). However, the confidence interval was wide and its lower limit was close to the null value, reflecting the small number of outcome events under this more restrictive definition. **Supplementary Figure 4** presents the Kaplan–Meier curve evaluating glaucoma risk in AA patients compared with non-AA controls in the US collaborative network, serving as cross-database validation. The association remained significant in the US collaborative network, with a hazard ratio of 2.11 (95% CI, 1.72–2.59).

### Subtype and stratified analyses

**Figure 3:**
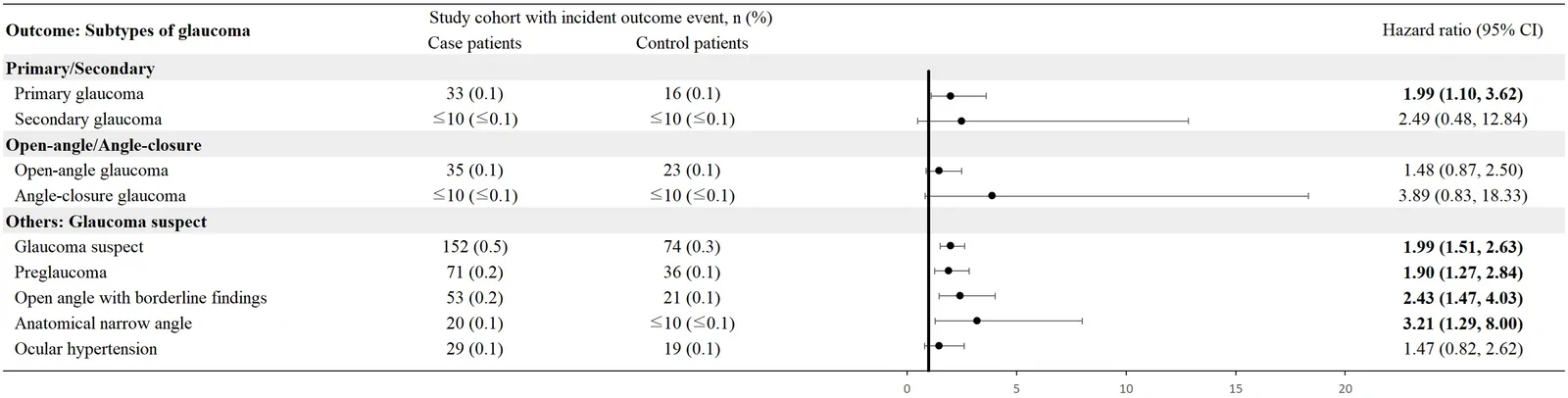
Subtypes of glaucoma in patients with AA.

**Figure 4:**
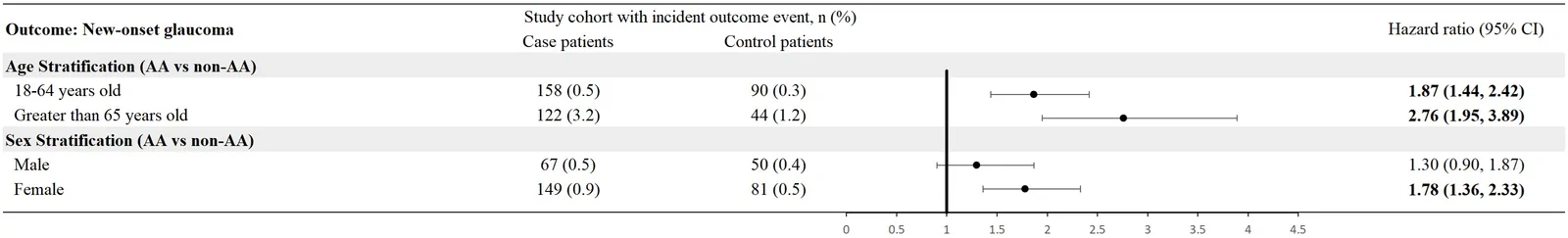
Stratification analysis based on age and sex.

As shown in **Figure 3**, patients with AA were associated with a significantly increased risk of primary glaucoma (HR: 1.99; 95% CI: 1.10–3.62; n=33 in the AA group vs n=16 in the control group). Elevated risks were also observed for early or suspected glaucoma categories, including preglaucoma (HR: 1.90; 95% CI: 1.27–2.84; n=71 vs n=36), open-angle glaucoma with borderline findings (HR: 2.43; 95% CI: 1.47–4.03; n=53 vs n=21), and anatomical narrow angle (HR: 3.21; 95% CI: 1.29–8.00; n=20 vs n ≤ 10). In contrast, other glaucoma subtypes did not demonstrate statistically significant associations. **Figure 4** presents the stratified analysis results. Among females with AA, the risk of new-onset glaucoma was 1.78-fold higher than in non-AA females (95% CI: 1.36–2.33), whereas no statistically significant association was observed in males (HR: 1.30; 95% CI: 0.90–1.87). Age-stratified analyses demonstrated elevated glaucoma risk in both the 18–64 and >65 year groups. Notably, patients aged >65 years had a 2.76-fold increased risk (95% CI: 1.95–3.89) compared with their matched controls.

## Discussion

This is a multicenter cohort study to investigate the long-term association between AA and glaucoma, utilizing a robust, large-scale database. The risk was predominantly observed in women and individuals over 65 years of age. Notably, the observed associations were more pronounced in early or suspected glaucoma categories, whereas findings for more established glaucoma subtypes were less consistent. This pattern suggests that the results may partly reflect increased detection of early or subclinical disease rather than confirmed glaucomatous optic neuropathy. Importantly, given the retrospective and observational nature of this study, the observed associations should not be interpreted as evidence of a causal relationship. Moreover, because glaucoma was not confirmed using intraocular pressure, optical coherence tomography, visual-field testing, or specialist review, the present data cannot determine whether the observed association corresponds to structural or functional glaucomatous damage.

Because hair follicles and the eye are both immune-privileged sites, the collapse of follicular immune privilege in AA may reflect a broader immune milieu associated with dysregulation at other protected tissues (6). AA is characterized by increased major histocompatibility complex expression around the hair follicle and activation of IFN-γ–producing cytotoxic CD8^+^ T cells (24), particularly NKG2D^+^CD8^+^ T cells (25). IFN-γ enhances antigen presentation and induces IL-15 expression through JAK1/JAK2–STAT1 signaling (26), whereas IL-15 promotes the survival, activation, and cytotoxic function of these T cells through JAK1/JAK3–STAT5 signaling, thereby sustaining a reciprocal inflammatory circuit (27, 28). Th1-polarized responses are central to alopecia areata pathogenesis, with IFN-γ-producing cytotoxic T cells driving hair follicle immune privilege collapse (2, 8). IL-23 is elevated and correlates with disease severity, though the downstream Th17 response shows variable activation across studies (29, 30). Additionally, Th2 skewing has been consistently observed in lesional tissue, suggesting complex multi-axis immune dysregulation (31).

Although glaucoma is primarily characterized as a chronic neurodegenerative optic neuropathy, intraocular inflammation is directly involved in several secondary forms, including uveitic glaucoma (32). A higher prevalence of T cell–mediated autoimmune diseases has also been reported among patients with primary open-angle glaucoma (13), and glaucoma has been proposed as a disorder in which adaptive immune responses may contribute to retinal neurodegeneration (33). Experimental and translational evidence suggests that age-related changes, together with mechanical and oxidative stress and persistent inflammation, can compromise the blood–retinal barrier (34–36). These alterations may enhance local antigen presentation, thereby promoting the infiltration of activated peripheral T cells into the retina or optic nerve head (37, 38). These infiltrating T cells may recognize stress-associated antigens, including heat-shock proteins expressed by injured retinal cells, and interact with retinal microglia and Müller glia (39–41). Subsequent glial activation may increase the local production of IFN-γ, TNF-α, IL-1β, reactive oxygen species, and complement-related mediators (42). Together, these processes could establish a sustained neuroinflammatory environment associated with mitochondrial dysfunction, impaired axonal transport, retinal ganglion cell apoptosis, and axonal degeneration (33, 43–45).

Notably, prior clinical studies have reported no significant differences in intraocular pressure between patients with alopecia areata and controls. For example, Esmer et al. conducted a cross-sectional study including patients with alopecia areata and matched healthy controls and found no significant differences in intraocular pressure or visual field parameters (14). Several factors may explain the discrepancy between their findings and ours. First, their study design was cross-sectional and based on a relatively small sample size, which limits the ability to assess long-term risk or incident disease. In contrast, our study employed a large-scale longitudinal cohort design with extended follow-up, enabling evaluation of new-onset glaucoma over time. Second, differences in study populations may contribute to the observed variation. The Esmer et al. cohort consisted of relatively young patients undergoing detailed ophthalmologic examination in a controlled clinical setting, whereas our study reflects a broader, real-world population across multiple healthcare systems. Third, glaucoma pathogenesis is increasingly recognized as multifactorial and not solely dependent on intraocular pressure elevation. Immune-mediated neuroinflammation, vascular dysregulation, and retinal ganglion cell susceptibility may contribute to glaucomatous damage independent of IOP. Therefore, it is plausible that systemic immune dysregulation in AA influences glaucoma risk through non–IOP-dependent mechanisms, which would not be detected in studies focusing primarily on cross-sectional ophthalmic measurements.

However, given the observational nature of this study and the use of administrative data, these proposed mechanisms should be considered hypothesis-generating, and the present findings should be interpreted as demonstrating an epidemiologic association rather than direct mechanistic evidence. The systemic Th1/Th17 polarization and cytotoxic T-cell activation observed in AA could theoretically lower the threshold for ocular immune dysregulation in individuals exposed to inflammatory, or pressure-related stress. However, this interpretation remains speculative because the present analysis did not include direct measures of ocular or systemic immune activity. It also lacked histopathological data, detailed retinal imaging, longitudinal intraocular pressure profiles, and assessments of blood–retinal barrier integrity. We were therefore unable to determine whether the observed association was mediated by IFN-γ–IL-15 signaling or by T-cell–driven mechanisms involving Th17 cells, cytotoxic CD8+ T cells, and heat-shock protein–reactive T cells. A potential contribution from glial activation or other immune pathways also could not be excluded. These mechanisms should not be interpreted as evidence that AA biologically causes glaucoma. A more cautious interpretation is that AA and glaucoma may share overlapping immune and neuroinflammatory pathways. Prospective studies integrating systemic immunophenotyping, ocular-fluid biomarkers, and standardized structural and functional ophthalmologic assessments are needed to evaluate this possibility.

In the current stratified analysis, we found that the risk was more pronounced in patients aged over 65 years. This finding highlights a potential synergistic effect between age-related immune changes and the systemic pathophysiology of AA. Age is a well-established risk factor for glaucoma, primarily mediated by a process known as immunosenescence. This aging of the immune system compromises the eye’s immune privilege, promoting a state of chronic low-grade inflammation, or para-inflammation, that makes the optic nerve more susceptible to damage (46). The weakened ocular microenvironment in older patients may therefore render them particularly vulnerable to the systemic immune dysregulation characteristic of AA, leading to a more pronounced risk of glaucoma. Additionally, the risk was significantly higher in female AA patients, with no statistically significant association observed in males. Previous studies indicated that the changes in the level of female sex hormones could affect IOP and vascular resistance, leaving the optic nerve more vulnerable to damage (47, 48). Furthermore, due to an anatomically narrower anterior chamber angle, women are at higher risk of developing angle-closure glaucoma (49). In addition, given that AA is a T cell–mediated autoimmune disease, and women generally exhibit stronger immune responses and higher susceptibility to autoimmune conditions than men (50, 51), the confluence of these hormonal, anatomical risk and immunological factors may predispose female AA patients to a heightened risk of glaucoma.

The stronger associations observed for glaucoma-suspect and borderline categories raised the possibility that the primary composite result was partly driven by early or provisional diagnostic coding. Nevertheless, in a sensitivity analysis excluding glaucoma-suspect conditions coded as H40.0, the association remained statistically significant (HR, 1.68; 95% CI, 1.01–2.78). This finding suggests that the overall association was not attributable solely to glaucoma-suspect diagnoses. However, the estimate was imprecise, with a wide confidence interval and a lower limit close to the null value, owing to the limited number of outcomes under the more restrictive definition. The result should therefore be considered supportive but not definitive. Moreover, despite the large sample size and consistency across sensitivity and validation analyses, the present study remains primarily descriptive. The analytical approach was based on propensity score matching and time-to-event comparisons using routinely collected electronic health record data. It therefore cannot determine the biological mechanism underlying the observed association or establish that immune dysregulation in AA directly contributes to glaucomatous neurodegeneration. The mechanistic pathways discussed below should consequently be regarded as biologically plausible hypotheses derived from prior experimental and clinical literature rather than mechanisms demonstrated by the present analysis.

However, several limitations warrant consideration. First, given the retrospective observational design, our findings are associative rather than causal, and unmeasured genetic, environmental, or lifestyle factors may contribute to both AA and glaucoma (52, 53). Second, outcome ascertainment was based entirely on EHR-derived ICD-10-CM codes without specialist adjudication or access to the clinical measurements required to confirm glaucoma. A single glaucoma-related code recorded after the index date was sufficient to define an outcome, and repeated coding was not required. Moreover, the primary composite outcome included glaucoma-suspect and other early or borderline diagnostic categories. Important ophthalmologic parameters, including intraocular pressure, optical coherence tomography–derived retinal nerve fiber layer thickness, visual-field testing, cup-to-disc ratio, and indices of functional impairment, were unavailable within the TriNetX platform. We therefore could not confirm glaucoma according to standardized clinical criteria, distinguish pre-perimetric from perimetric disease, or assess disease progression. Accordingly, this study evaluates glaucoma-related diagnostic coding rather than clinically adjudicated glaucoma. Misclassification is therefore possible, and the estimates may also reflect differences in screening intensity, referral patterns, or diagnostic coding practices between cohorts. These limitations are particularly important when interpreting the findings for glaucoma-suspect, preglaucoma, and borderline categories. Third, residual confounding could exist, as confounding bias caused by comedications are common in real-world studies, and should be prudently interpreted (54). For instance, corticosteroids might elevate intraocular pressure and precipitate steroid-induced glaucoma (55, 56); however, granular exposure data (including dose, route, duration, timing, and adherence) were not available within the TriNetX platform, limiting precise adjustment for treatment effects. To address this concern, we performed additional analyses excluding individuals with systemic corticosteroid use and conducted an alternative propensity score matching model incorporating corticosteroid use as a covariate, both of which yielded consistent results. Nevertheless, medication exposure could not be modeled as a time-dependent variable, and the temporal relationship between corticosteroid use and glaucoma onset could not be fully delineated. Therefore, residual confounding related to corticosteroid exposure may persist. Nonetheless, prior studies that adjusted for steroid use have demonstrated independent associations between autoimmune conditions and glaucoma, supporting the robustness of our main findings (13). Fourth, granular information regarding AA severity and clinical phenotype was unavailable. The TriNetX platform does not provide validated Severity of Alopecia Tool scores, percentage of scalp involvement, alopecia totalis, alopecia universalis, ophiasis-pattern disease, disease duration, relapse frequency, or treatment response. We were therefore unable to distinguish limited patchy AA from extensive, chronic, or treatment-refractory disease or to evaluate whether the association with glaucoma-related diagnostic coding varied according to AA severity. This is clinically relevant because the magnitude and persistence of systemic immune activation may differ substantially across AA phenotypes. Accordingly, the present estimate should be interpreted as an average association across a heterogeneous AA population, and it cannot establish a severity-dependent or dose–response relationship. Future studies incorporating standardized dermatologic severity assessments and longitudinal treatment-response data are needed to determine whether patients with more extensive or active AA have a different ocular risk profile. Fifth, patients with AA may have more frequent healthcare utilization and clinical encounters, which could increase the likelihood of ophthalmic evaluations and subsequent detection of glaucoma, thereby introducing surveillance bias. This concern is particularly relevant for diagnostic categories such as glaucoma suspect, preglaucoma, open-angle with borderline findings, and anatomical narrow angle, which may be more sensitive to screening intensity and diagnostic practices rather than representing definite glaucomatous optic neuropathy. In addition, the use of a control cohort defined by routine health examinations (ICD-10-CM code Z00) may further contribute to a “healthy user” effect, as these individuals may differ systematically in healthcare-seeking behavior. Detailed data on ophthalmic visits or eye examination frequency were not available within the TriNetX platform, precluding direct adjustment for healthcare utilization intensity. To partially address surveillance bias, we performed an active-comparator analysis using patients with psoriasis, another chronic inflammatory skin disease associated with ongoing medical care and repeated healthcare contact. The association was modestly attenuated but remained statistically significant when AA was compared with psoriasis (HR, 1.74; 95% CI, 1.43–2.12), suggesting that the overall excess risk of glaucoma was not entirely attributable to comparison with a relatively healthy general examination cohort. Nevertheless, this analysis does not eliminate surveillance bias. Patients with AA and psoriasis may differ in healthcare utilization, specialist referral patterns, treatment exposure, disease severity, and the likelihood of undergoing ophthalmic evaluation. Furthermore, the active-comparator analysis assessed the composite glaucoma outcome and was not separately performed for individual glaucoma subtypes. It therefore cannot determine whether the associations observed for glaucoma suspect, preglaucoma, open-angle glaucoma with borderline findings, or anatomical narrow angle were independent of differential screening and diagnostic intensity. Because these categories often reflect early or screening-detected abnormalities rather than established glaucomatous optic neuropathy, their associations remain particularly susceptible to outcome ascertainment bias. Accordingly, the psoriasis comparison strengthens the robustness of the overall finding but should not be interpreted as fully resolving surveillance bias, especially for glaucoma-suspect outcomes. Sixth, although TriNetX is multicenter, participating sites are predominantly in North America and Europe and the cohort is largely of White or Black/African ancestry, limiting generalizability to Asian populations and other healthcare systems. Moreover, as the analytic cohort comprised adults (≥18 years) exclusively, its applicability to pediatric and adolescent populations cannot be assumed. Seventh, selection and data heterogeneity intrinsic to the TriNetX network may remain. Because the platform captures encounters only from participating medical centers, mild or unrecognized disease and out-of-network events may be under-ascertained, potentially underestimating incidence and risk.

In conclusion, AA was associated with a higher subsequent rate of glaucoma-related diagnostic coding in TriNetX electronic health record network. However, the outcomes were not confirmed using intraocular pressure measurements, optical coherence tomography, visual-field testing, or specialist adjudication. The findings therefore represent an epidemiologic association with recorded glaucoma-related diagnoses rather than clinically confirmed glaucomatous optic neuropathy. They should not be interpreted as evidence of causality or a demonstrated biological link. Prospective studies using standardized ophthalmologic examinations and longitudinal structural and functional measurements are needed to validate the association.

## Data Availability

The data analyzed in this study is subject to the following licenses/restrictions: Data in this study were retrieved from TriNetX Research Network. All data available in the database were administrated by the TriNetX platform. Detailed information can be retrieved at the official website of the research network (https://trinetx.com). Requests to access these datasets should be directed to TriNetX research network (https://trinetx.com).
