# Supplementary material for: Alopecia areata patients are associated with increased risk of glaucoma: a multicenter, retrospective cohort study
Source: Front Immunol. 2026 Aug 12;17:1870857. doi: 10.3389/fimmu.2026.1870857 (PMC13506891; doi:10.3389/fimmu.2026.1870857)
Supplement: Supplementary file 1 [file Table1.docx]

**Supplementary Tables**

**Table S1.** Utilized proxy codes ^a^

| **Description** | **ICD-10-CM codes** |
| --- | --- |
| *Study population* |  |
| Alopecia areata | ICD-10-CM L63 |
| *Major outcome event* |  |
| Glaucoma | ICD-10-CM: H40-H42 |
| *Subsets of glaucoma* |  |
| Primary glaucoma | ICD-10-CM: H40.11, H40.2, Q15.0 |
| Secondary glaucoma | ICD-10-CM: H40.3-H40.6 |
| Open-angle glaucoma | ICD-10-CM: H40.1 |
| Angle-closure glaucoma | ICD-10-CM: H40.2 |
| Glaucoma suspect | ICD-10-CM: H40.0 |
| Preglaucoma | ICD-10-CM: H40.00 |
| Open angle with borderline findings | ICD-10-CM: H40.01-H40.02 |
| Anatomical narrow angle | ICD-10-CM: H40.03 |
| Ocular hypertension | ICD-10-CM: H40.05 |
| *Covariates and other definitions* |  |
| Essential hypertension | ICD-10-CM: I10 |
| Hyperlipidemia | ICD-10-CM: E78.5 |
| Diabetes mellitus | ICD-10-CM: E08-E13 |
| Chronic ischemic heart disease | ICD-10-CM: I25 |
| Systemic lupus erythematosus | ICD-10-CM: M32 |
| Sjögren's syndrome | ICD-10-CM: M35.0 |
| Ankylosing spondylitis | ICD-10-CM: M45 |
| Rheumatoid arthritis | ICD-10-CM: M05 |
| Conjunctivitis | ICD-10-CM: H10 |
| Iridocyclitis | ICD-10-CM: H20 |
| Persons with potential health hazards related to socioeconomic and psychosocial circumstances | ICD-10-CM: Z55-Z65 |
| Mental and behavioral disorders due to psychoactive substance use | ICD-10-CM: F10-F19 |
| Encounter for general examination | ICD-10-CM: Z00 |
| Comedications |  |
| Corticosteroids for systemic use | ATC code: H02 |
| Epinephrine | RxNorm: 3992 |
| Anticholinergics | ATC code: R03BB |
| Tricyclic antidepressants | VA code: CN601 |

^a^ICD-10-CM: International Classification of Diseases, Tenth Revision, Clinical Modification

**Table S2.** Description of all applied sensitivity analysis models

| **Models** | **Description** |
| --- | --- |
| **Applying various proxy-based alopecia areata (AA) definition** | |
| Algorithm 1 | Only patients with the record of the record of ICD-10-CM codes of L63 (alopecia areata) with inpatient record were included as AA group in this model. |
| Algorithm 2 | Only patients with the record of the record of ICD-10-CM codes of L63 (alopecia areata) with prescription of systemic corticosteroid (ATC code:H02) were included as AA group in this model. |
| **Applying different wash-out period after index date** | |
| 24 months/36 months after index date | Outcome event occurs within the set wash-out period will be excluded from further analysis. In all sub-analyses, the follow-up period was set as 15 years. |
| 5 years /10 years /15 years after index date | Only the outcome event occurs within the set follow-up time will be counted. In all sub-analyses, the wash-out period was set as 3 months. |
| **Applying different matching covariates** | |
| Crude model | Hazard ratio was calculated based on the population without propensity score matching |
| Matching model 1 | Matching covariates include age and sex |
| **Applying various active comparator** |  |
| Comparing with psoriasis patients | The AA cohort was defined using the same criteria as in the primary analysis. The active-comparator cohort consisted of patients with psoriasis identified by ICD-10-CM code L40 and without any diagnosis of AA. Patients younger than 18 years, those with any history of malignancy, individuals documented as deceased, and those with glaucoma diagnosed before or on the index date were excluded from both cohorts. The cohorts were matched 1:1 using the same sociodemographic, clinical, and healthcare-utilization proxy covariates as in the primary analysis. The outcome was new-onset glaucoma defined by ICD-10-CM codes H40–H42, with a 3-month washout period and 15-year follow-up. |

**Table S3.** Risk of glaucoma in AA vs Non-AA control cohorts after considering systemic corticosteroid use^a^

|  | **Hazard ratio (95% CI)** |
| --- | --- |
| Adding systemic corticosteroid uses in covariates of propensity score matching model^b^ | 1.866 (95% CI, 1.584,2.197) |
| Excluding individuals with the record of systemic corticosteroid use in both alopecia areata and non- alopecia areata cohorts | 2.262 (95% CI, 1.632,3.135) |

**^a^** 1:1 propensity score matching was performed. The matching covariates included in this analysis includes age, sex, race, body mass index, lifestyle (mental and behavioral disorders due to psychoactive substance use), socioeconomic status (persons with potential health hazards related to socioeconomic and psychosocial circumstances) as well as other comorbidities (including hypertension, hyperlipidemia, diabetes mellitus) and systemic corticosteroid uses.

**Supplementary figures**

**Figure S1.** Cumulative probability of glaucoma in AA patients and non-AA patients

**Legends:** The dataset used in this analysis was from Global collaborative network, and the outcome event in this model was defined as glaucoma (ICD-10-CM: H40–H42).

**Figure S2.** Cumulative probability of glaucoma in AA patients and non-AA patients

**Legends:** The dataset used in this analysis was from Global collaborative network, and the outcome event in this model is set as glaucoma (ICD-10-CM: H40-H42), excluding angle closure glaucoma (ICD-10-CM: H40.2) and secondary glaucoma (ICD-10-CM: H40.3-H40.6)

**Figure S3.** Cumulative probability of glaucoma in AA patients and non-AA patients

**Legends:** The dataset used in this analysis was from Global collaborative network, and the outcome event in this model is set as glaucoma (ICD-10-CM: H40-H42), not including glaucoma suspect (ICD-10-CM: H40.0).

**Figure S4.** Cross-database validation: cumulative probability of glaucoma in AA patients and non-AA patients

**Legends:** The dataset used in this analysis was from US collaborative network, and the outcome event in this model is set as glaucoma (ICD-10-CM: H40-H42).
